# Low birth weight of institutional births in Cambodia: Analysis of the Demographic and Health Surveys 2010-2014

**DOI:** 10.1371/journal.pone.0207021

**Published:** 2018-11-08

**Authors:** Chhorvann Chhea, Por Ir, Heng Sopheab

**Affiliations:** School of Public Health at the National Institute of Public Health, Tuol Kork District, Phnom Penh, Cambodia; B P Koirala Institute of Health Sciences, NEPAL

## Abstract

**Background:**

Low birth weight (LBW), an important risk factor for early childhood mortality and morbidity, is a major public health concern in developing countries including Cambodia. This study examined the prevalence of LBW across provinces in Cambodia and changes over time, and identified the factors associated with such condition.

**Methods:**

We used children datasets from Cambodia Demographic and Health Survey (CDHS) 2010 and 2014. There were 3,522 children and 4,991 children in both surveys. Maps illustrating provincial variation in LBW prevalence were constructed. Then, multivariate analyses were conducted to assess factors independently associated with LBW in CDHS 2014.

**Results:**

LBW prevalence remained stable between 2010 and 2014, at around 7.0% 95% CI: 5.8–8.1). all institutional births, but within significant variation across provinces. Factors independently associated with LBW included mother’s no education compared with those whose mothers had secondary or higher education (AOR = 1.6, 95% CI: 1.0–2.6), babies born to mothers with < 4 antenatal care (ANC) visits during the pregnancy compared with those whose mothers had at least 4 ANC visits (AOR = 2.0, 95% CI: 1.5–2.8). Also, first-born babies were at greater risk of LBW compared with second-born babies (AOR = 1.4, 95% CI: 1.0–2.0).

**Conclusion:**

The study points to key sub-populations at greater risk and regions where LBW is particularly prevalent. Programs should target provinces where LBW prevalence remains high. Illiterate women, especially those pregnant for the first time should be the program priority. The current national program policy, which recommends that pregnant women have ≥ 4 ANC visits during pregnancy should be further reinforced and implemented. Program design should consider ways to communicate the importance of making the recommended number of ANC visits among women with no formal education.

## Background

Low birth weight (LBW) has been defined as a birth weight of less than 2,500 grams (or 5.5 pounds), irrespective of gestational age [1]. LBW is a strong risk factor for neonatal and early childhood mortality and morbidity [2]. It is estimated that LBW babies are 20 times more likely to die in the first year than babies with normal weight [1]. Recent studies show that LBW also increases the risk for non-communicable diseases, such as diabetes and cardio-vascular disease, later in life [3]. LBW continues to be a major public health problem worldwide, particularly in developing countries. Globally in 2013, nearly 22 million newborns (16% of all newborns) had LBW [4]. However, the prevalence of LBW varies considerably across regions and within countries. It was estimated that 97% of LBW occurs in low- and middle-income countries [1], and especially among the most vulnerable populations, including the poor living in remote areas. Among regions, prevalence of LBW is highest in South Asia, at 28%, followed by Sub-Saharan Africa, 13%, Latin America and the Caribbean, 9%, and 6% in East Asia and the Pacific [1, 5, 6].

LBW prevalence in Cambodia is higher than the average in East Asia and the Pacific as a whole. According to the World Bank, the prevalence of LBW in Cambodia in 2000 was 11%. Prevalence declined to around 8% of all live births in 2005 [7], and has remained at about the same level in 2010 and 2014 [7, 8]. The Cambodia Demographic and Health Survey (CDHS) 2014 shows great variation in LBW by place of residence; LBW is higher in rural areas (8%) compared with urban areas (6%), and varies across the country’s regions, from 5% in Battambang/Pailin provinces to over 12% in Siem Reap province [9]. WHO data (2014) indicated that LBW contributed to about 3.0% of the total number of deaths in Cambodia [10]. These data suggest that LBW is still an important public health problem in Cambodia, and further reduction of LBW prevalence should be a national policy priority.

LBW is associated with a number of risk factors. These include age, education, mother’s marital status, and employment, level of household wealth, ethnic origin and body mass index (BMI) of the mothers [11–14]. A recent analysis of DHS data in 10 developing countries [14] found that older mothers (age 35–49) had significantly higher risk of delivering LBW babies compared with younger mothers, while other studies have found a higher prevalence of LBW among babies born to younger mothers [15, 16]. Some studies found LBW to be associated with prenatal maternal depression and anxiety [17–19]. Such antenatal mental condition is prevalent in Cambodia and their relation to LBW has been established [20, 21]. Other studies also demonstrated that early child marriage and adolescent pregnancy contribute to LBW [22, 23]. Other related risk factors are included pre-pregnancy weight, parity, history of prior LBW, gestational weight gain and caloric intake, general morbidity and illnesses, malaria, maternal HIV infection cigarette smoking, alcohol consumption, and history of LBW and prematurity [11, 14, 24]. For example, a study by Zheng et al. among 92,641 mothers in Okinawa showed that maternal smoking was significantly associated with LBW in all age groups, while Coutinho et al. found that smoking beyond the fourth month of pregnancy was a risk factor for LBW, irrespective of the number of cigarettes smoked per day [12, 25]. Mother’s level of hemoglobin, and baby’s birth interval are also associated with LBW [11, 12]. The number of antenatal care visits during pregnancy is also the important risk factor of LBW [12–14, 26, 27]. Kader et al. found attending fewer than 4 ANC visits to be a maternal risk factor for LBW, whereas the other studies did not specify a threshold number of ANC visits, but only an inadequate number of ANC visits. The relative importance of these factors, however, is time-bound and country-specific.

Reducing the prevalence of LBW in Cambodia requires a better understanding of its geographic distribution and associated risk factors, to help identify sub-populations at greater risk and priority geographic regions. To the authors’ knowledge, no in-depth study of the factors associated with LBW in Cambodia has been conducted to date. Therefore, the study addresses this knowledge gap based on CDHS 2010 and 2014 data. The objectives of this study are twofold: first, to describe the prevalence of LBW across CDHS 2010 and 2014 by province using GIS mapping; and second, to explore key individual and household-level factors associated with LBW. Better understanding of such factors may help develop policies and programs with more effective strategies and interventions to reduce prevalence of LBW, thereby contributing further to the reduction of neonatal mortality in Cambodia.

## Methods

We used children data from CDHS 2010 and CDHS 2014 to map the distribution of LBW by province, and used CDHS 2014 to conduct logistic multivariate analyses to explore the factors associated with LBW among babies born in the 5 years preceding the survey.

The CDHS is a population-based nationally representative survey in which participants were selected using a two-stage stratified cluster sampling design. In the first stage, a defined number of enumeration areas (EAs or clusters) stratified by urban-rural were selected with probability proportional to EA size. In the second stage, a defined number of households (generally 25–30) were randomly selected from the listed households in each EA with simple random selection. Eligible women age 15–49 in the selected households were invited for interviews, and data about the women and their children born in the past 5 years preceding the survey were collected [9].

**Fig 1:**
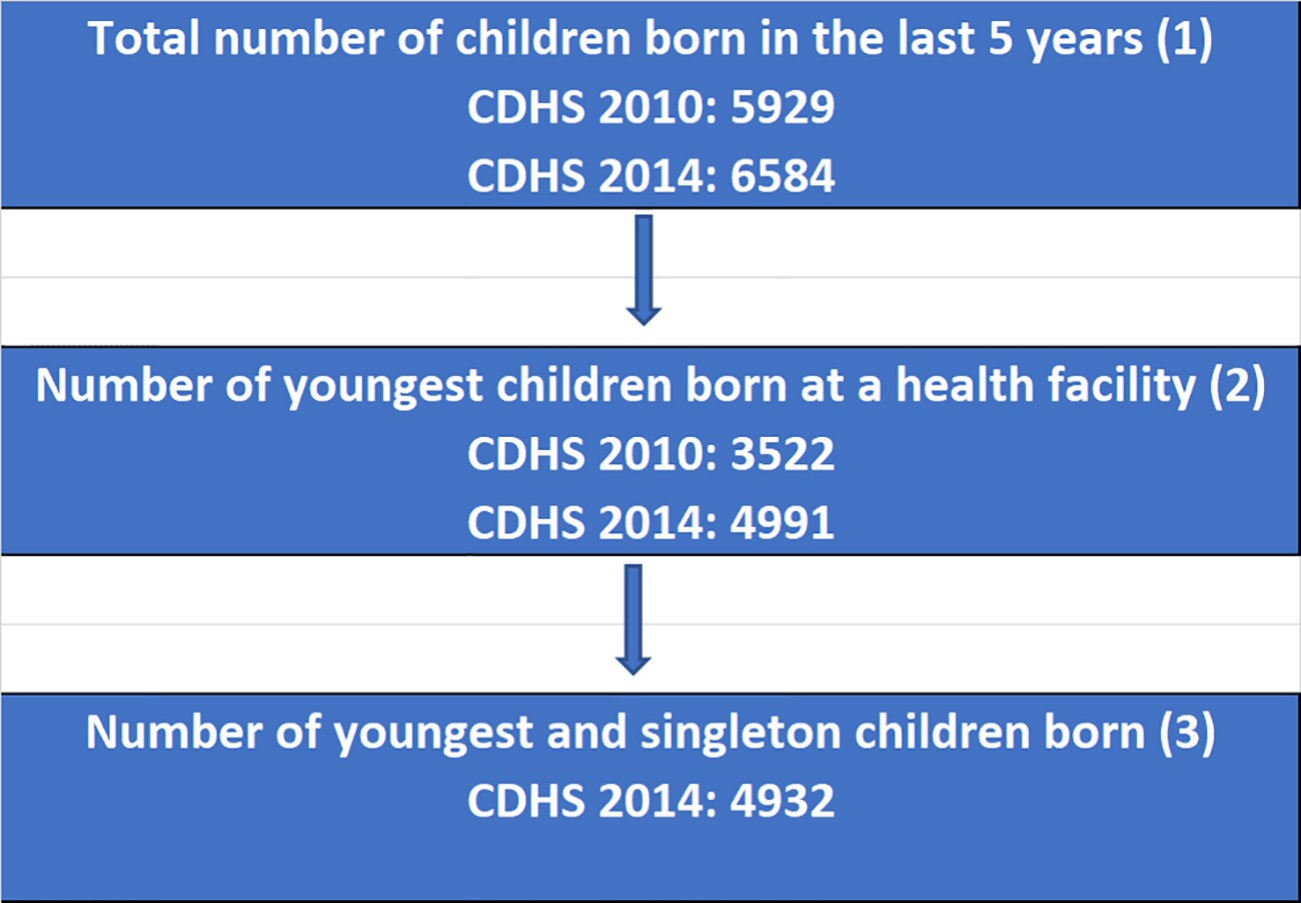
Sample selection diagram.

Fig 1 illustrates the derivation of the analytical samples for this study. In total, there were 5,929 live births in the 5 years preceding the 2010 CDHS and 6,584 live births in the 5 years preceding the 2014 CDHS (Panel 1). We limited the analysis to last births because the CDHS collected data on ANC only for women’s last pregnancy. Due to potential inaccuracy of birth weight taken at home, we restrict our analysis to live births that occurred at health facilities, which represent 53% and 82% of all live births in the 2010 and 2014 surveys, respectively. This process resulted in a final sample size of 3,522 children from the 2010 CDHS and 4,991 children from the 2014 CDHS (Panel 2). Finally, we further restricted our analysis to singleton babies, because multiple births are strongly associated with premature birth and LBW. Multiple births accounted for only 1.2% of all last live births that occurred at health facilities. Therefore, the final sample for multivariate logistic regression included 4,932 youngest and singleton children born at health facilities in the CDHS 2014 (Panel 3).

### Outcome variable

The outcome variable of interest is the prevalence of low birth weight among the youngest singleton children born at a health facility. Based on the definition of LBW (weight at birth of less than 2,500 grams), children were classified into 2 groups: non-LBW (birth weight ≥ 2,500 grams, coded 0) or LBW (birth weight < 2,500 grams, coded 1).

### Explanatory variables

The study included the following explanatory variables: household and mother’s characteristics and behaviors, access to health care during pregnancy, maternal health status, and baby’s characteristics. Maternal age at the time of giving birth was divided into 3 categories: <18 years; 18–34 years; and 35–49 years. Marital status was classified as currently married/in union or formerly married. Level of education was defined as: no schooling; primary school; and secondary and higher. Current employment status was classified into: no job or not working; agricultural jobs or self-employed; professional or technical or sales jobs; and other types of jobs, which included manual labor and unskilled jobs. Whether the mother smoked cigarettes was recoded as yes (coded 1) or no (coded 0). Mothers’ perceived problems in accessing health care is a dichotomous variable, coded 0 if the mother reported no perceived barrier and coded 1 if the mother reported 1 or more barriers (distance, money, and waiting time). Number of ANC visits was recoded as a binary variable: mothers who made fewer than 4 visits were coded 0, and those who made 4 or more visits were coded 1. Whether the mother received ANC with nutritional counseling and health insurance coverage were both recoded as either yes or no. Mother’s anemia was defined by her hemoglobin level: severe (0–69 g/dl); moderate (70–99 g/dl); mild (100–119 g/dl); and not anemic (>120 g/dl). Because of few observations, the severe anemia category was grouped together with moderate anemia. Mother’s BMI was calculated as weight-to-height ratio (kg/m^2^), and further categorized as: underweight (<18.5 kg/m^2^); normal or healthy weight (18.5–24.9 kg/m^2^); and overweight (≥24.9 kg/m^2^).

Birth order was classified as: first child, second and third child, and fourth+ child. Birth interval was defined by the number of years following the first-born child, and children were then classified in 4 groups: first child; <2 years; 2–3 years; and >3 years. The sex of the child was recorded as boy or girl. Household wealth status was measured based on the ownership of durable assets, using principal component analysis. The constructed wealth index values were then equally split into quintiles: lowest; second; middle; fourth; and highest. Place of residence was classified as rural or urban. Since the number of CDHS domains (provinces) is too many for meaningful analysis (19), we regrouped them for regression analysis into 4 geographical regions, as administratively defined in Cambodia: Northwest; Northeast; Central; and South.

### Statistical analysis

Key socioeconomic and demographic characteristics of the sample in CDHS 2014—youngest children born in health facilities in the past 5 years preceding the survey and their mothers—were described and expressed in frequency and percentage. Outliers were rechecked for outcome variable and explanatory variables though they were cleaned in the original CDHS children dataset. The analyses were performed in STATA V14 (Stata Corp 2015, College Station, TX) considering sampling weights, cluster effect and urban rural stratification to adjust for the complex sampling design of the CDHS.

The prevalence of LBW and its distribution across the 19 domains (provinces) was estimated, using CDHS 2010 and CDHS 2014. The results were then used to construct maps illustrating provincial variation in the prevalence of LBW and trends over time, using QGIS software V 2.18.7 (GIS Development Team). Significant difference was assessed between 2010 and 2014, using Chi-square test; and between provincial LBW in CDHS 2010 and CDHS 2014, using logistic regression.

We used bivariate analyses to assess the association between prevalence of LBW and household characteristics, mother’s characteristics and behaviors, access to health care during pregnancy, maternal health status, and baby’s characteristics. Multivariate logistic regression was used to predict the independent factors associated with LBW. Odds ratios with 95% confidence intervals were reported. Finally, we restricted our multivariate logistic regression to singleton babies; therefore, the sample for multivariate logistic regression included 4,932 youngest and singleton children born at health facilities in the CDHS 2014. All covariates significantly associated with LBW at the p value ≤ 0.20 in bivariate analyses were included in the multivariate logistic regression. Also, interested covariates were included in the multivariate regression regardless their significant levels.

Multicollinearity among the following variables was assessed: geographical regions, residence, household wealth index, mother’s age at the time of giving birth, anemia, BMI, and child’s birth order. No serious collinearity between variables was detected (the highest correlation coefficient was <0.6). In the final model, 9 covariates were kept. These covariates were: mother’s age at the time of giving birth; education level; reported number of ANC visits; anemia level; BMI level; child’s sex; residence (urban-rural); geographical regions; and wealth index.

### Ethical approval

The CDHS 2014 was approved by the National Ethics Committee for Health Research (Ref: 056 NECHR), Cambodia and the Institutional Review Board (IRB) of ICF in Rockville, Maryland, USA. The CDHS data are publicly accessible and were made available to us upon request to the DHS Program, ICF. Written consents were obtained from all participants before the interview with participants in the CDHS.

## Results

### Description of the sample

Table 1 describes the socioeconomic and demographic characteristics of the 4,991 youngest children born in health facilities in the 5 years preceding the survey, and the characteristics of their mothers. Eighty-three percent of children were born in rural areas. Most of the children were singleton births; and only 1.2% was twins. Among the sample, 1.5% died after birth. Their average weight at birth was 3,100 grams. Nearly 90% of the mothers were age 18–34 at the time of childbirth, and 95% were currently married or in union. Eleven percent of the mothers had no schooling, 51% had a primary education, and 38% had a secondary or higher education. Only 1% of the mothers smoked cigarettes. The great majority of the mothers (81%) attended at least 4 antenatal care (ANC) visits during pregnancy, 15% 2–3 visits, 2% 1 visit, and 1% no ANC visit. Just over half (56%) of the mothers were not anemic, whereas 39% had mild anemia, and 6% moderate anemia, respectively. Only 0.1% of them had severe anemia (Table 1).

**Table 1:** Socioeconomic and demographic characteristics of the final study samples, CDHS 2014.

Variables	N = 4,991	
	Frequency	%
***Mother’s characteristics***		
**Age at the time of giving birth**		
<18	114	2.2
18–34	4,454	89.3
35–49	423	8.5
**Marital status**		
Currently married/in union	4,741	95.0
Formerly married	250	5.0
**Education**		
No schooling	556	11.1
Primary school	2,522	50.5
Secondary school/higher	1,913	38.3
**Current employment status**		
No job/not working	1,256	25.2
Agricultural jobs and self-employed	1,546	31
Professional, technical and sales jobs	2,128	42.6
Others (manual labor and unskilled jobs)	60	1.2
**Smoking cigarettes**		
No	4,935	98.9
Yes	55	1.1
**Perceived problems in accessing health service**		
No barrier	1,558	31.2
≥ 1 barrier (distance/money/waiting time)	3,433	68.8
**Number of ANC visits**		
No ANC visit	66	1.3
1 visit	107	2.1
2–3 visits	747	15
≥ 4 visits	4,059	81.3
Don’t know/missing	12	0.2
**ANC with nutrition counseling**		
No	567	11.4
Yes	4,358	87.3
No information	66	1.3
**Health insurance coverage**		
No	4,117	82.5
Yes	874	17.5
Anemia* **(N = 3,277)**		
Severe	4	0.1
Moderate	182	5.6
Mild	1,265	38.6
No anemia	1,826	55.7
Body Mass Index* **(N = 3,017)**		
Underweight	326	10.8
Normal weight	2,161	71.6
Overweight	437	14.5
Obese	93	3.1
***Children’s characteristics***		
**Birth status**		
Single birth	4,932	98.8
Twin birth	59	1.2
**Birth order**		
1^st^ child	1,918	38.4
2^nd^ or 3^rd^ child	2,386	47.8
4^th^ child or higher	687	13.8
Birth interval* **(N = 4,973)**		
1^st^ child	1,918	38.6
<2 years	357	7.2
2–3 years	712	14.3
>3 years	1,986	39.9
**Sex**		
Boy	2,544	51.0
Girl	2,447	49.0
**Status after birth**		
Not alive	76	1.5
Alive	4,915	98.5
**Average birth weight (SD)**	**3,100 grams (SD = 9.3)**	
Range	700–6000 grams	
*Household characteristics*		
**Place of residence**		
Urban	836	16.8
Rural	4,155	83.2
**Wealth index**		
Lowest	936	18.8
Second	955	19.1
Middle	989	19.8
Fourth	968	19.4
Highest	1,144	22.9

* The total sample in some variables were less than the total final study sample (N = 4991).

The distribution of the sample by geographic domain (province or group of province) in CDHS 2010 and CDHS 2014 is presented in **Appendix 1.** In CDHS 2010, Phnom Penh (14%), Kampong Cham (11%), and Kandal (11%) accounted for the 3 largest samples, while Odar Meanchey, Kratie, Preah Vihear/Stung Treng, and Modulkiri/Rattanakiri were least represented, at only about 1% each, among the 19 domains. This distribution changed slightly in CDHS 2014. Phnom Penh (10%) and Kampong Cham (14%) were the 2 largest samples, while Kandal fell to 7% of the total sample in CDHS 2014. Odar Meanchey, Kratie, Preah Vihear/Stung Treng, and Modulkiri/Rattanakiri remained the 4 smallest samples.

### Descriptive analysis of LBW prevalence in 2010 and 2014 by province

**Fig 2:**
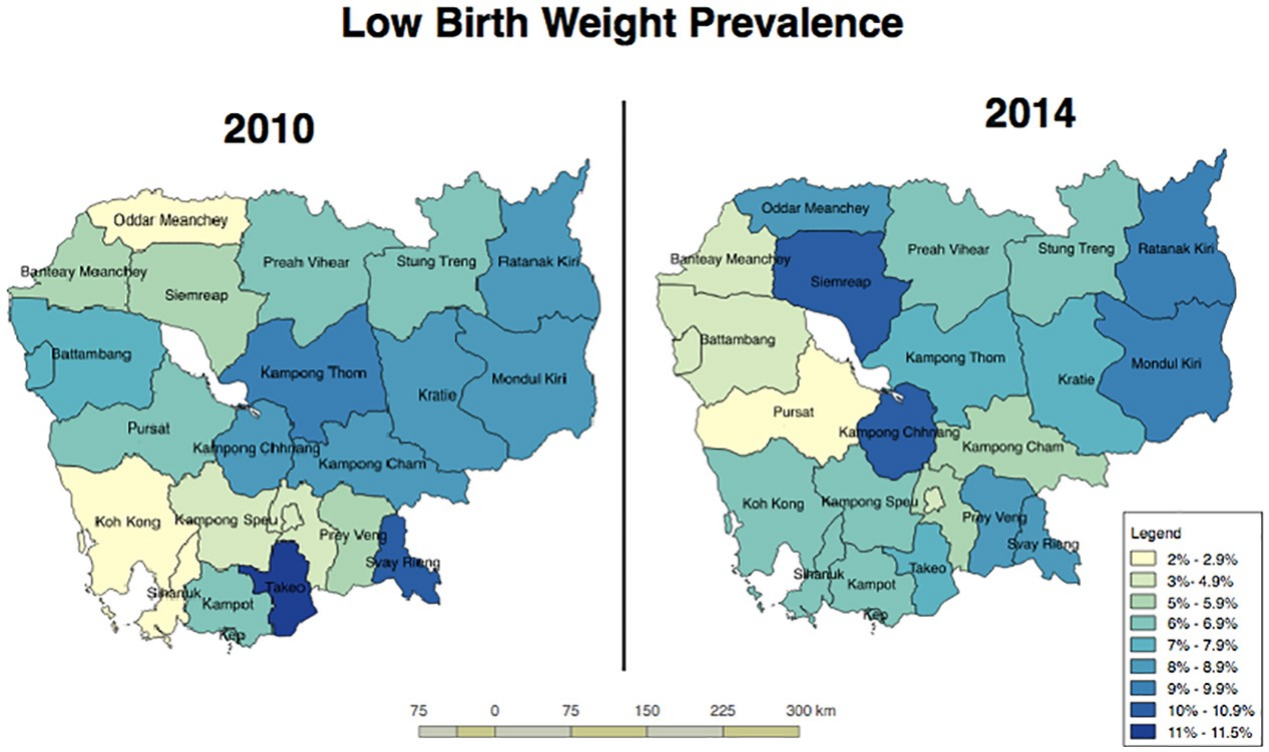
Distribution of LBW prevalence in CDHS 2010 and 2014 by province.

Overall, the prevalence of low birth weight among the samples (last births at health facilities) remained stable between 2010 and 2014, at about 7.0% (95% CI: 5.8–8.1) and 6.9% (95% CI: 6.1–7.9) respectively. However, the variation by province varied across the 2 surveys. Fig 2 shows the distribution of LBW prevalence in CDHS 2010 and CDHS 2014 by province, with the darker color indicating higher prevalence of LBW.

**Appendix 2** presents the data on LBW prevalence for the 19 domains. In 2010, the highest LBW prevalence was in Takeo province (12%) and Svay Rieng (10%), and the lowest was in Sihanouk Ville/Koh Kong (3.5%) and Odar Meanchey (2.5%). This distribution changed between 2010 and 2014. In 2014, the highest prevalence of LBW was in Siem Reap (11%) and Kampong Chhnang (10%), an increase compared with 2010 (7% for Siem Reap and 8% for Kampong Chhnang). However, the increase in both provinces was not statistically significant. LBW prevalence in Svay Rieng and Takeo declined slightly in 2014, to 9% and 10%, respectively, but not statistically significant.

### Bivariate analysis of LBW prevalence in 2014 by key explanatory variables

Table 2 presents the prevalence of LBW by key explanatory variables, including household and mother’s characteristics and behaviors, access to health care during pregnancy, health insurance coverage, maternal health status, and child’s characteristics.

**Table 2:** Prevalence of LBW among singleton babies born in health facilities by key explanatory variables, CDHS 2014.

Variables	CDHS 2014 (N = 4,932)			
	Total number of births	% with LBW	95% CI	P value
***Mother’s characteristics***				
**Age at the time of giving birth**				
<18	114	9.0	[5.0, 15.7]	0.142
18–34	4,399	6.2	[5.3, 7.2]	
35–49	419	8.7	[5.8, 12.8]	
**Marital status**				
Currently married/in union	4,685	6.4	[5.5, 7.3]	0.447
Formerly married	247	7.9	[4.6, 13.2]	
**Education**				
No schooling	544	9.3	[6.5, 13.1]	0.073
Primary school	2,489	5.9	[4.8, 7.2]	
Secondary school/higher	1,899	6.4	[5.2, 7.8]	
**Current employment status**				
No job/not working	1,233	6.6	[5.1, 8.6]	0.493
Agricultural jobs and self-employed	1,526	7.3	[5.8, 9.0]	
Professional, technical and sales jobs	2,113	5.8	[4.6, 7.3]	
Others (manual labor and unskilled jobs)	60	5.3	[1.5, 16.9]	
**Smoking cigarettes**				
No	4,880	6.4	[5.6, 7.4]	0.937
Yes	51	6.1	[1.9, 17.9]	
**Perceived problems in accessing health service**				
No barrier	1,548	6.4	[5.1, 8.1]	0.989
≥ 1 barrier (distance/money/waiting time)	3,384	6.4	[5.5, 7.5]	
**Number of ANC visits during pregnancy**				
<4 visits	917	9.9	[7.4, 13.1]	**< 0.001**
≥4 visits	4,016	5.6	[4.8, 6.6]	
**ANC with nutritional counseling**				
No	559	8.9	[5.9, 13.1]	0.045
Yes	4,308	6.0	[5.2, 7.0]	
No information	66	12.5	[5.8, 24.9]	
**Health insurance coverage**				
No	4,079	6.1	[5.3, 7.1]	0.171
Yes	853	7.9	[5.7, 10.9]	
**Anemia (N = 3,243)**				
Severe/moderate	185	7.5	[3.8, 14.4]	0.864
Mild	1,249	6.8	[5.0, 9.1]	
No anemia	1,809	6.2	[5.0, 7.7]	
**Body mass index (N = 2,984)**				
Underweight	323	7.3	[4.5, 11.8]	0.159
Normal weight	2,140	6.8	[5.5, 8.3]	
Overweight	428	5.1	[2.9, 8.8]	
Obese	93	1.1	[0.3, 3.9]	
*Child’s characteristics*				
**Birth order**				
1^st^ child	1,918	8.5	[7.1, 10.2]	**< 0.001**
2^nd^ or 3^rd^ child	2,357	4.7	[3.6, 6.0]	
4^th^ child or higher	657	6.7	[4.6, 9.7]	
**Birth interval**				
1^st^ child	1,918	8.5	[7.1, 10.2]	**< 0.001**
<2 years	352	7.1	[4.2, 11.7]	
2–3 years	701	6.0	[4.0, 8.9]	
>3 years	1,961	4.4	[3.5, 5.6]	
**Sex**				
Boy	2,523	6.1	[5.1, 7.2]	0.348
Girl	2,409	6.8	[5.6, 8.2]	
***Household characteristics***				
**Place of residence**				
Urban	828	4.4	[3.0, 6.5]	**0.037**
Rural	4,104	6.8	[5.9, 7.9]	
Geographical regions*				
Northwest	1,243	5.9	[4.31, 7.9]	0.602
Northeast	296	8.1	[6.3, 10.7]	
Central	1,573	6.2	[4.8, 8.0]	
South	1,820	6.7	[5.4, 8.3]	
**Wealth index**				
Lowest	915	8.1	[6.0, 10.9]	**0.069**
Second	943	7.8	[5.9, 10.1]	
Middle	981	6.4	[4.8, 8.6]	
Fourth	955	5.7	[4.2, 7.8]	
Highest	1,138	4.6	[3.2, 6.6]	

**Northwest**: Banteay Meanchey, Pursat, Siem Reap, Odor Meanchey, Battambang/Pailin, **Northeast:** Kratie, Preah Vihear/Stung Treng, Modulkiri/Rattanakiri, **Central:** Kampong Chhnang, Kampong Thom, Kampong Cham, Kandal, Phnom Penh, **South:** Takeo, Kampot/Kep, Koh Kong/Sihanouk Ville

Prevalence of LBW among children born to mothers with no schooling, at 9%, was higher than among those born to mothers with a primary education or a secondary or higher education, at 6% each, although the difference was not statistically significant. The prevalence of LBW did not vary by mother’s age, marital status, current employment status, smoking status, perceptions of problems in accessing health service, and health insurance coverage. Yet, LBW prevalence among children born to mothers attending less than 4 ANC visits during pregnancy (10%) was significantly higher than for those born to mothers attending 4 or more ANC visits (6%). The prevalence of LBW was lower among babies born to mothers who received nutritional counseling during the ANC visits (6%) compared with those who did not (9%). There was no significant difference in the prevalence of LBW by mother’s anemia levels or body mass index (BMI). First-born babies had a higher LBW prevalence (9%) than second- and third-born babies (5%) and fourth-born or higher (7%). There were no differences in the prevalence of LBW between boy and girl babies.

Prevalence of LBW was substantially higher in rural areas (7%) than urban areas (4%) but did not differ much by region: Northwest (6%), Northeast (8%), Central (6%), and South (7%). The prevalence of LBW among children born to mothers in the lowest and second household wealth quintiles (each about 8%) was higher than for those in the middle quintile (6%), the fourth quintile (6%), and highest quintile (5%). This difference was not statistically significant (P value = 0.069) (Table 2)

### Results from logistic regression analysis

Table 3 summarizes the results of the logistic regression of factors associated with LBW among singleton children born in health facilities, based on the 2014 CDHS. For comparison purposes, the unadjusted odds ratios (OR) and 95% confidence interval (CI) are also presented (the first 2 columns of Table 3). The OR indicates that the following factors were significantly associated with LBW: mother’s education level, number of ANC visits during pregnancy, place of residence, household wealth index, birth order, and birth interval. Mothers with no schooling had higher odds of having babies with LBW compared with those with a secondary or higher education (OR = 1.7, 95% CI: 1.1–2.4). Mothers who attended fewer than 4 ANC visits during their last pregnancy had 1.8 times higher odds of LBW compared with women having 4 or more ANC visits (OR = 1.8, 95% CI: 1.3–2.6). Children born to mothers in rural areas had 1.6 times higher odds of LBW than those born to urban mothers (OR = 1.6, 95% CI: 1.0–2.5). Children in the highest household quintile had lower odds of LBW compared with those in the lowest quintile (OR = 0.5, 95% CI: 0.3–0.9). First-born children and children born within 1 year from a prior birth were each about twice as likely to have LBW. Mother’s marital status, current employment, smoking status, perception of barriers to health care access, health insurance coverage, and receipt of nutritional counseling were not correlated with LBW and therefore were excluded from the multivariate analysis. Other factors were also not significantly associated with LBW, such as geographical region, mother’s age at the time of giving birth, BMI, and anemia, but these variables were still included in the multivariate model.

**Table 3:** Factors associated with LBW among singleton babies born in health facilities, results of the univariate and multivariate logistic regressions, CDHS 2014.

Variables	Total, N = 4,932		Total, N = 3,941	
	OR	95% CI	AOR^a^	95% CI
**Mother’s age of the time of giving birth**				
<18	1	-	1	-
18–34	1.0	0.5–2.2	0.7	0.3–1.7
35–49	0.7	0.4–1.1	1.4	0.8–2.4
**Mother’s marital status**				
Currently married/in union	1	-	-	-
Formerly married	1.3	0.7–2.3	-	-
**Mother’s level of education**				
No schooling	1.7**	**1.1–2.4**	1.6*	**1.0–2.6**
Primary school	1.0	0.7–1.3	0.9	0.6–1.3
Secondary school/higher	1	-	1	-
**Mother’s current employment**				
No job	1.3	0.3–4.5	-	-
Agricultural jobs and self-employed	1.4	0.4–5.1	-	-
Others (manual labor and other jobs)	1.1	0.3–4.0	-	-
Professional, technical and sales jobs	1	-	-	-
**Mother smoking cigarettes**				
No	1	-	-	-
Yes	1.1	0.3–3.5	-	-
**Perceived problem in accessing health services**				
No barrier	1	-	-	-
≥ 1 barrier (distance/money/waiting)	1	0.7–1.3	-	-
**Number of ANC visits during pregnancy**				
< 4 visits	1.8**	**1.3–2.6**	2.0***	**1.5–2.8**
≥ 4 visits	1	-	1	-
**ANC with nutritional counseling**				
Yes	1	-	-	-
No	1.5	1.0–2.4	-	-
No information	2.2	1.0–5.2	-	-
**Health insurance coverage**				
No	1.3	0.9–1.9	-	-
Yes	1	-	-	-
**Mother’s anemia**				
Severe/moderate	1.2	0.6–2.6	1.2	0.7–2.1
Mild	1.1	0.7–1.6	1.0	0.7–1.4
No anemia	1	-	1	-
**Mother’s Body Mass Index**				
<18.5 (underweight)	1.7	0.9–3.5	1.5	0.8–2.5
18.5–24.9 (normal weight)	1.6	0.9–2.8	1.2	0.8–1.8
≥ 25 (Overweight)	1	-	1	-
**Child’s birth order**				
1^st^ child	1.9***	**1.4–2.6**	1.4*	**1.0–2.0**
4^th^ child or higher	1.5	0.9–2.4	1.2	0.7–2.0
2^nd^ or 3^rd^ child	1	-	1	-
**Child’s birth interval**				
1^st^ child	2.0***	**1.5–2.8**	-	-
<2 years	1.7	1.0–2.9	-	-
2–3 years	1.4	0.8–2.2	-	-
>3 years	1	-	-	-
**Child’s sex**				
Boy	1	-	1	-
Girl	1.1	0.9–1.5	1.2	0.9–1.7
**Residence**				
Urban	1	-	1	-
Rural	1.6*	**1.0–2.5**	1.2	0.6–2.2
**Geographical region**^**1**^				
Northwest	1	-	1	-
Northeast	1.4	0.9–2.2	0.7	0.4–1.2
Central	1.1	0.7–1.6	1.0	0.6–1.6
South	1.2	0.8–1.7	1.0	0.6–1.6
**Wealth index**				
Lowest	1	-	1	-
Second	1.0	0.6–1.5	0.8	0.5–1.3
Middle	0.8	0.5–1.2	0.8	0.5–1.2
Fourth	0.7	0.4–1.1	0.9	0.5–1.4
Highest	0.5*	**0.3–0.9**	0.6	0.3–1.1

*** p<0.001,

** p<0.01,

* p<0.0;

AOR = Adjusted Odds Ratio. **Northwest**: Banteay Meanchey, Pursat, Siem Reap, Odor Meanchey, Battambang/Pailin, **Northeast:** Kratie, Preah Vihear/Stung Treng, Modulkiri/Rattanakiri, **Central:** Kampong Chhnang, Kampong Thom, Kampong Cham, Kandal, Phnom Penh, **South:** Takeo, Kampot/Kep, Koh Kong/Sihanouk Ville

In the multivariate logistic model (the last 2 columns of Table 3), factors significantly and independently associated with LBW included mother’s education level, number of ANC visits, and birth order of the children. Again, mother’s education was a strong predictor of LBW among singleton children born at health facilities. Children born to mothers with no education were more likely to have LBW compared with those whose mother had a secondary or higher education with adjusted OR (AOR) = 1.6 (95% CI: 1.0–2.6) after controlling other factors. Mothers reported attending fewer than 4 ANC visits during their last pregnancy remained an independent predictor of having babies with LBW (AOR = 2.0, 95% CI: 1.5–2.8) compared with mothers attending 4 or more ANC visits, after controlling for other factors that influence birth weight. Finally, being a first-born child was still strongly associated with LBW (AOR = 1.4, 95% CI: 1.0–2.0) compared with being the second child. The effects of rural-urban residence and household wealth index were no longer statistically significant after controlling for other covariates in the model (Table 3).

## Discussion

This study described provincial variations in the prevalence of low birth weight in Cambodia and changes in prevalence over time, based on the CDHS conducted in 2010 and 2014. The study also explored key individual and household-level factors associated with LBW among singleton live births that occurred in health facilities, using data from CDHS 2014. The prevalence of LBW resulted from our analysis remained stable at around 7% of all singleton live births in health facilities in both CDHS 2010 and CDHS 2014, consistent with the findings presented in the 2 survey reports which included all live births regardless place of birth and number of babies [8, 9]. This figure is much lower than the global average of 16%, but slightly higher than the average of 6% in the East Asia and Pacific region [1, 6]. The distribution of LBW prevalence by province across the 2 survey rounds showed great variation, with high rates in some provinces, in particular Siem Reap and Kampong Chhnang, where prevalence increased considerably between surveys (although the increases were not statistically significant).

Results from multivariate logistic regression showed that mother’s educational level, number of ANC visits during the last pregnancy, and birth order were significantly and independently associated with LBW in Cambodia. These findings are consistent with other studies that document the importance of mother’s educational level and number of ANC visits. The findings that mothers with no schooling (illiterate mothers) were 1.6 times more likely to have a LBW baby compared with those with a secondary or higher education is similar to findings of 3 studies in other countries that illiteracy of mothers is a risk factor for LBW [13, 14, 28].

The study’s finding that mothers who attended fewer than 4 ANC visits during their last pregnancy were twice as likely to have babies with LBW, compared with mothers who attended 4 or more ANC visits, is also in line with other studies elsewhere [29–31]. Mother with lower ANC visits may leave pregnant mothers without proper weight monitoring, nutrition counselling and proper access to supplements of iron and folic acid pills during the pregnancy. Therefore, the finding supports the current national policy of Cambodia, which recommends at least 4 ANC visits during pregnancy [32]. This finding that the first child is more likely to have LBW compared with the second or third child has not been seen in any study except for a study showing that first and fourth children tend to have lower birth weight than second- and third-born children [33].

While a study in several developing countries found that rural residence and poverty are risk factors for LBW [14], the present study found that these factors, although significantly associated with LBW in bivariate analysis, were no longer associated with LBW after adjusting for other covariates in the multivariate logistic regression.

This study has 3 major limitations. First, it was limited to explanatory variables for which data were collected and reported in CDHS 2010 and CDHS 2014. Data on many potential correlates of low birth weight or risk factors were not available, such as past history of premature births or LBW, early child marriage, adolescent pregnancy, maternal HIV infection and maternal morbidity and illnesses during pregnancy, including non-communicable diseases such as hypertension, diabetes and mental disorders.

Second, the available data were measured at the time of survey, not during pregnancy or at the time of delivery. While many of these variables, such as household wealth status and residence, are unlikely to have changed, others such as mother’s employment status, marital status, BMI, and anemia may differ between the time of pregnancy or delivery and the time of survey. We estimated maternal age at the time of giving birth by deducting the child’s age from the mother’s age. For BMI, the analysis did not include women who were pregnant at the time of survey but who had a live birth in the 5 years preceding the survey. Another concern was the challenge in dealing with missing data of outcome variable of LBW. However, the concern is not significant since less than 1% of the data was missing (CDHS 2014).

Third, the analysis was restricted to children born at a health facility. It excluded home births because babies born at home may not have been weighed or appropriately weighed. This might bias the results, particularly since children not weighed or not appropriately weighed at birth are more likely to be born to less educated or poorer mothers who may be less aware of the importance of maternal health care or lack the financial resources to afford the minimum recommended 4 ANC visits, and therefore more likely to have LBW children.

## Conclusions

The findings suggest provincial variations in the two surveys and three risk factors. Reducing the prevalence of LBW in Cambodia is possible, but challenging. Interventions to reduce LBW should target provinces where prevalence remains relatively high, or has increased in recent years, such as Siem Reap and Kampong Chhnang. Such targeting should be guided by further in-depth studies on the factors related to LBW and maternal health care.

Furthermore, the study shows that mother’s educational level; number of ANC visits during the last pregnancy, and child’s birth order are significantly associated with LBW or risk factors of LBW. These results generally confirm the findings of research in other countries. The findings that illiterate mothers have higher risk of having LBW babies and that first-born children are at greater risk than subsequent births suggest that women with no formal education, especially those who are pregnant for the first time, should be a priority for programs to reduce the incidence of LBW. The findings strongly support and reconfirm the current national program policy in Cambodia that recommends pregnant women to have at least 4 ANC visits during pregnancy. This policy should be further reinforced and implemented, with consideration for finding effective ways to reach illiterate women with information and support for antenatal care.

## Supporting information

S1 Appendix Sample distribution by domain (province or group of provinces) in CDHS 2010 and 2014. (DOC)

S2 Appendix Prevalence of low birth weight in the 19 domains in CDHS 2010 and 2014. (DOC)

S1 File Data file (Cdhs lbw 2014 hf_V13). (DTA)
