# Supplementary material for: Low birth weight of institutional births in Cambodia: Analysis of the Demographic and Health Surveys 2010-2014
Source: PLoS One. 2018 Nov 8;13(11):e0207021. doi: 10.1371/journal.pone.0207021 (PMC6224106; doi:10.1371/journal.pone.0207021)
Supplement: S1 Appendix — (DOC) [file pone.0207021.s001.doc]

**Appendix 1: Sample distribution by domain (province or group of provinces) in CDHS 2010 and 2014**

| **Variables** | | **CDHS 2010 (N = 3,566)** | | **CDHS 2014 (N = 4,991)** | |
| --- | --- | --- | --- | --- | --- |
|  |  | **Frequency** | **%** | **Frequency** | **%** |
| **Provinces** | |  |  |  |  |
| 1 | Banteay Meanchey | 134 | 3.8 | 197 | 4.0 |
| 2 | Kampong Cham | 379 | 10.6 | 680 | 13.6 |
| 3 | Kampong Chhnang | 159 | 4.5 | 200 | 4 |
| 4 | Kampong Speu | 196 | 5.5 | 329 | 6.6 |
| 5 | Kampong Thom | 131 | 3.7 | 206 | 4.1 |
| 6 | Kandal | 408 | 11.4 | 343 | 6.9 |
| 7 | Kratie | 46 | 1.3 | 104 | 2.1 |
| 8 | Phnom Penh | 504 | 14.1 | 509 | 10.2 |
| 9 | Prey Veng | 230 | 6.4 | 372 | 7.5 |
| 10 | Pursat | 104 | 2.9 | 193 | 3.9 |
| 11 | Siem Reap | 322 | 9.0 | 356 | 7.1 |
| 12 | Svay Rieng | 110 | 3.1 | 181 | 3.6 |
| 13 | Takeo | 280 | 7.9 | 294 | 5.9 |
| 14 | Odar Meanchey | 51 | 1.4 | 103 | 2.1 |
| 15 | Battambong/Pailin | 225 | 6.3 | 413 | 8.3 |
| 16 | Kampot/Kep | 118 | 3.3 | 190 | 3.8 |
| 17 | Sihanoukville/Koh Kong | 86 | 2.4 | 127 | 2.5 |
| 18 | Preah Vihear/Stung Treng | 41 | 1.1 | 102 | 2.0 |
| 19 | Mondulkiri/Rattanakiri | 40 | 1.1 | 93 | 1.9 |
