# Supplementary material for: Low birth weight of institutional births in Cambodia: Analysis of the Demographic and Health Surveys 2010-2014
Source: PLoS One. 2018 Nov 8;13(11):e0207021. doi: 10.1371/journal.pone.0207021 (PMC6224106; doi:10.1371/journal.pone.0207021)
Supplement: S2 Appendix — (DOC) [file pone.0207021.s002.doc]

**Appendix 2: Prevalence of low birth weight in the 19 domains in CDHS 2010 and 2014**

| **Province** | **CDHS 2010 (N = 3,566)** | |  | **CDHS 2014 (N = 4,991)** | |
| --- | --- | --- | --- | --- | --- |
|  | **% of LBW** | **[95% CI]** |  | **% of LBW** | **[95% CI]** |
| Banteay Meanchey | 5.9 | [3.2,10.6] |  | 5.6 | [3.4,9.1] |
| Kampong Cham | 8.5 | [4.9,14.4] |  | 5.9 | [3.5,9.9] |
| Kampong Chhnang | 7.9 | [4.7,13.0] |  | 10.1 | [6.8,14.9] |
| Kampong Speu | 3.6 | [1.4,9.0] |  | 6.5 | [3.7,11.3] |
| Kampong Thom | 8.9 | [4.8,15.9] |  | 7.4 | [4.5,12.1] |
| Kandal | 5.1 | [2.6,9.8] |  | 5.5 | [3.0,10.1] |
| Kratie | 8.9 | [4.1,18.4] |  | 7.7 | [4.6,12.6] |
| Phnom Penh | 5.1 | [3.3,7.9] |  | 4.9 | [2.7,8.7] |
| Prey Veng | 5.9 | [3.1,11.0] |  | 9.2 | [5.8,14.4] |
| Pursat | 6.5 | [3.6,11.2] |  | 2.2 | [0.8,5.8] |
| Siem Reap | 6.9 | [4.1,11.5] |  | 11.1 | [7.0,17.1] |
| Svay Rieng | 10.1 | [5.0,19.4] |  | 9.9 | [7.1,13.7] |
| Takeo | 11.9 | [6.6,20.4] |  | 8.6 | [5.7,12.9] |
| Odar Meanchey | 2.5 | [1.1,5.8] |  | 8.0 | [5.4,11.8] |
| Battambong/Pailin | 7.7 | [4.5,12.9] |  | 4.7 | [2.6,8.3] |
| Kampot/Kep | 6.4 | [2.9,13.3] |  | 6.4 | [3.6,11.1] |
| Sihanoukville/Koh Kong | 3.5 | [1.6,7.6] |  | 6.4 | [3.7,10.9] |
| Preah Vihear/Stung Treng | 6.6 | [2.4,16.5] |  | 6.9 | [3.9,11.9] |
| Mondulkiri/Rattanakiri | 8.0 | [4.8,13.0] |  | 9.9 | [6.6,14.6] |
